# P-458. Factors Associated with Completion of High-Resolution Anoscopy in a Cohort of Transgender Women – Findings from a Mixed-Methods Study

**DOI:** 10.1093/ofid/ofae631.658

**Published:** 2025-01-29

**Authors:** Omar Harfouch, Darren Whitfield, Tural Mammaldi, Megan E Mansfield, Rahwa Eyasu, Phyllis Bijole, Emade Ebah, Meredith Zoltick, Ashley Davis, Tina Liu, Miriam Jones, Rachel Silk, David Sternberg, Habib Omari, Shyamasundaran Kottilil, Henry Masur, Sarah Kattakuzhy, Elana S Rosenthal

**Affiliations:** University of Maryland Baltimore - Institute of Human Virology, Baltimore, Maryland; University of Maryland Baltimore, Baltimore, Maryland; University of Maryland Baltimore -School of Social Work, Baltimore, Maryland; University of Maryland Baltimore - Institute of Human Virology, Baltimore, Maryland; Institute for Human Virology (IHV), University of Maryland School of Medicine, Washington, District of Columbia; HIPS.org, Washington, District of Columbia; Institute for Human Virology (IHV), University of Maryland School of Medicine, Washington, District of Columbia; University of Maryland Baltimore - Institute of Human Virology, Baltimore, Maryland; Institute for Human Virology (IHV), University of Maryland School of Medicine, Washington, District of Columbia; National Institutes of Health - Critical Care Medicine Department, Durham, North Carolina; HIPS, Washington DC, District of Columbia; University of Maryland, Washington, DC; University of Maryland Baltimore - Institute of Human Virology, Baltimore, Maryland; University of Maryland Baltimore, Baltimore, Maryland; University of Maryland Baltimore - Institute of Human Virology, Baltimore, Maryland; Critical Care Medicine Department, National Institutes of Health (NIH), Bethesda, Maryland; Institute for Human Virology (IHV), University of Maryland School of Medicine, Washington, District of Columbia; Institute for Human Virology (IHV), University of Maryland School of Medicine, Washington, District of Columbia

## Abstract

**Background:**

Guidelines recommend anal cancer screening in transgender women (TGW) – particularly those living with HIV (LWH) - using anal cytology followed by high-resolution anoscopy (HRA) in those with abnormal results. This study examines factors associated with completion of HRA in TGW with abnormal cytology.

**Figure:**
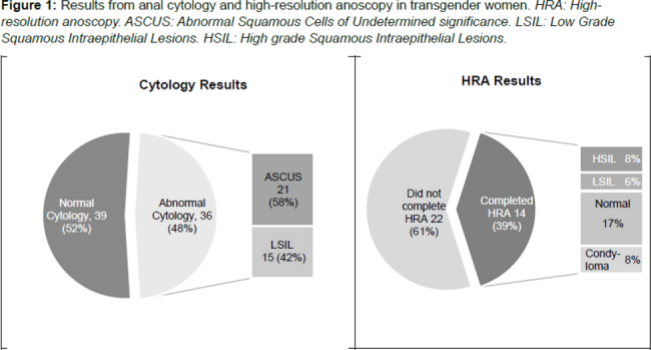

**Methods:**

We recruited 80 TGW using convenience sampling from 4/2021 – 9/2023. We collected survey data, serum samples, and anal swabs for cytology and HPV testing. TGW with abnormal cytology were scheduled for off-site HRA. Non-completion of HRA was defined as being scheduled for HRA but not completing it within 3 months of cytology result. We conducted semi-structured interviews with 8 TGW who completed HRA and 8 who didn’t complete HRA. We used chi-square tests and the weaved approach to compare factors associated with HRA completion from quantitative and qualitative data.

**Figure:**
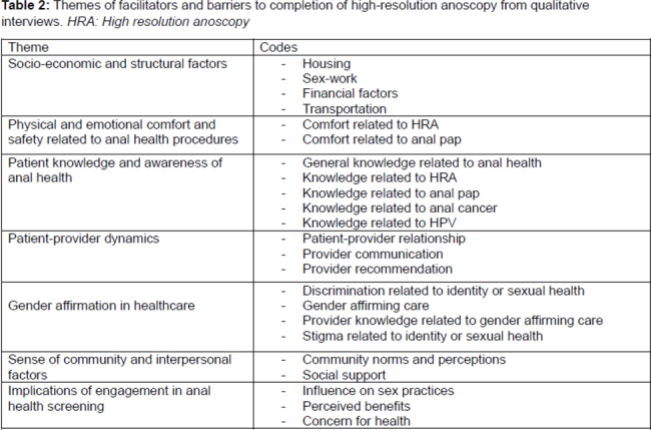

**Results:**

Of 75 TGW with anal cytology collected, 48% had abnormal results and of those 39% completed HRA (Figure 1). Of 36 TGW with abnormal cytology 97% were black, 61% were unstably housed, 78% were LWH. In the last year, 48% engaged in transactional sex and 69% in receptive anal sex (Table 1). HRA completion was associated with being employed (*p=0.01*) and, among TGW LWH, an undetectable HIV viral load (*p=0.01*) (Table 1). Qualitative data identified patient-provider trust as a facilitator to HRA completion and experiences of stigma or discrimination related to gender identity as a barrier to HRA completion (Table 2). Mixed-methods analysis revealed that socio-economic challenges, including housing, and financial factors – notably health insurance - were a barrier to HRA completion (Table 3).

**Figure:**
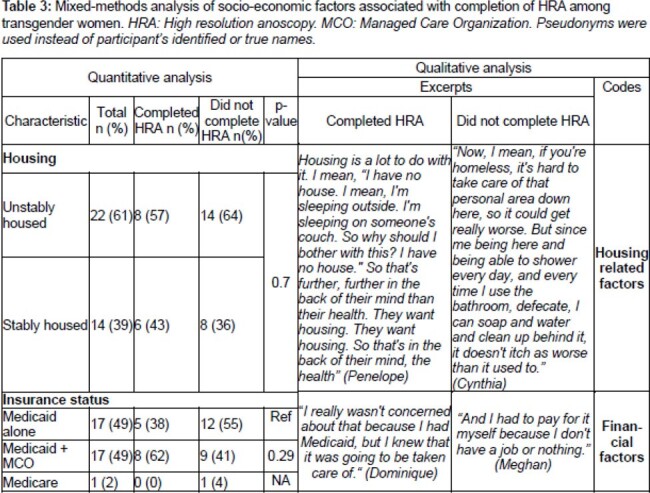

**Conclusion:**

In a cohort of TGW, we found high rates of abnormal anal cytology and low rates of HRA completion, highlighting missed opportunities for anal cancer prevention. Interventions aimed at improving anal cancer screening and linkage to HRA for TGW must be intentional about eschewing stigma and creating environments affirming of gender identity, while addressing barriers to care due to prevalent socioeconomic challenges. Additionally, TGW LWH with a detectable HIV viral load may require further targeted interventions to improve both the HIV care continuum and the anal cancer screening cascade.

**Figure:**
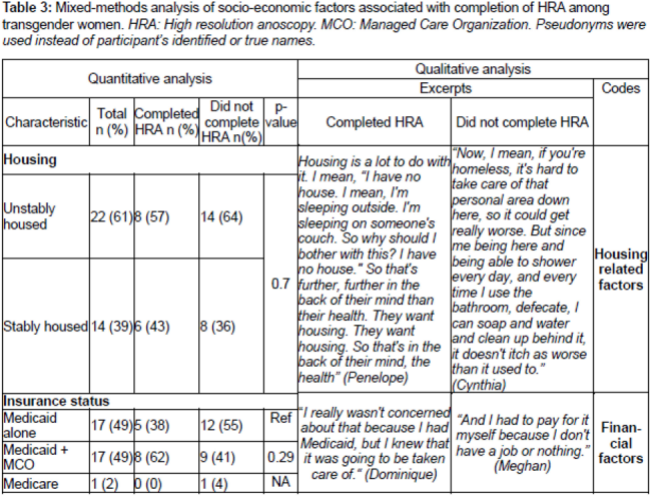

**Disclosures:**

**Shyamasundaran Kottilil, MD PhD**, CDA Foundation: Advisor/Consultant|Gilead Sciences: Advisor/Consultant|Orsobio: Advisor/Consultant|Orsobio: Stocks/Bonds (Private Company)|Red Queen Therapeutics: Advisor/Consultant **Elana S. Rosenthal, MD**, Gilead Sciences: Grant/Research Support|Merck: Grant/Research Support

